# Editor’s Choice Articles in the Manufacturing Processes and Systems Section: Emerging Directions in Advanced Manufacturing

**DOI:** 10.3390/ma19163420

**Published:** 2026-08-12

**Authors:** Abdollah Saboori

**Affiliations:** Integrated Additive Manufacturing Center (IAM@POLITO), Department of Management and Production Engineering, Politecnico di Torino, Corso Duca degli Abruzzi 24, 10129 Torino, Italy; abdollah.saboori@polito.it

## 1. Introduction and Scope

Manufacturing processes and systems are evolving from empirical process selection toward integrated, data-driven, and application-oriented engineering of materials, components, and functions. Additive manufacturing (AM) remains central to this transition by enabling the layer-wise fabrication of complex geometries from digital models and expanding the design space for mass customization, topology optimization, internal channels, lattice structures, and part consolidation that are challenging or in some cases impossible using conventional technologies [1,2]. For metallic materials, however, industrial performance strongly depends on more than geometric freedom and other important aspects such as processing condition, energy source–material interaction, thermal history, solidification, phase transformation, internal and surface quality, and post-processing rout, which can play a pivotal role in the final properties [3,4]. As a consequence, laser-based AM directed energy deposition and powder bed fusion processes have therefore shifted from rapid prototyping tools to manufacturing routes that require rigorous control of process–structure–property relationships [5,6].

In recent years, one of the key advances in AM has been the shift from open-loop parameter optimization to intelligent, closed-loop manufacturing. In fact, physics-based simulation, statistical learning, and artificial intelligence are combined with high-speed optical and thermal imaging, acoustic sensing, photodiodes, melt pool monitoring, layer-wise imaging, and X-ray computed tomography to detect anomalies, predict quality, and find the best processing window [7]. Moreover, it is well documented that the use of machine learning (ML) in AM has shifted from generating correlations between input parameters and density or melt pool geometry to supporting feature attribution, uncertainty-aware prediction, and process map generation [8]. The main objective is an adaptive manufacturing system where in situ data update the digital representation of the process and trigger corrective action during the process and qualification, moving towards digital twin and autonomous control [9].

In parallel with process digitalization, materials innovation has also been considered significantly. Extensive research is focused on feedstock preparation, alloy design, microstructure control, heat treatment, and process conditions as a coupled design problem. For instance, some efforts have been made to develop crack-resistant aluminum alloys, tailored tool steels, lightweight magnesium and titanium alloys, functionally graded or multi-materials, and metal matrix composites [10,11]. AM of ceramics is advancing through direct ink writing, vat photopolymerization, and related routes. In contrast, functional nanomaterials and surface-engineered systems are expanding manufacturing applications in energy, electronics, tribology, sensing, and biomedicine. This convergence of manufacturing science with computational thermodynamics, materials informatics, and multiscale characterization is accelerating the development of materials specifically designed for additive and hybrid processing rather than merely adapted from conventional production [12].

Hybrid manufacturing is another emerging approach for merging the design freedom and material efficiency of AM with the dimensional accuracy, surface integrity, and productivity of subtractive and finishing operations. Integrated additive–subtractive platforms, robotic deposition and machining cells, repair and remanufacturing workflows, advanced joining, and localized surface modification are receiving growing attention [13,14]. These methods are very important because as-built components frequently require machining, polishing, heat treatment, hot isostatic pressing, coating, or other post-processing before they can satisfy demanding requirements for fatigue resistance, corrosion behavior, tribological performance, and certification [15]. Therefore, precision grinding, interface engineering, and joining remain integral to advanced manufacturing systems for different sectors including aerospace, energy, tooling, semiconductor, and biomedical.

Another priority is moving from laboratory-scale demonstrations to repeatable, certifiable, and economically viable production. This transition needs robust feedstock specifications, standardized testing, statistically valid process windows, equipment-to-equipment comparability, and qualification strategies that account for spatial variability and build-to-build uncertainty [16,17]. The digital thread is becoming increasingly important in this regard by connecting design intent, material selection, machine parameters, monitoring data, post-processing history, inspection results, and service performance within a traceable information framework [18,19]. This kind of integration supports qualification by evidence, while also enabling predictive maintenance, root-cause analysis, and more rapid transfer of processes between machines and production sites. However, the industrial adoption of these approaches remains constrained by proprietary data formats, limited benchmark datasets, uncertain model generalizability, cybersecurity concerns, and the difficulty of validating AI models under changing materials and operating conditions [7,20,21].

Sustainability in part production or repair via AM technologies is also becoming a defining research track. The potential benefits of advanced manufacturing include lightweight design, reduced material waste, repair of high-value components, localized production, shorter supply chains, and more efficient use of critical materials [22,23]. In order to benefit from these advantages, energy intensity, powder and feedstock production, emissions, recyclability, process yield, equipment utilization, and end-of-life strategies must be evaluated across the full life cycle. Emerging work therefore connects process optimization with life-cycle assessment, circular-economy principles, recycled feedstocks, remanufacturing, and resource-efficient production [24]. Within this rapidly developing landscape, the selected Editor’s Choice Articles provide a representative overview of current directions in the Manufacturing Processes and Systems Section, spanning metallic and ceramic AM, hybrid manufacturing, joining, tribology, precision grinding, durability, and digital process design.

## 2. Advanced Manufacturing: Key Challenges and Future Directions

Advanced manufacturing is changing how complex parts are designed, produced, and improved. Technologies such as AM, advanced welding, rapid sintering, laser processing, and thermomechanical treatment make it possible to create components that would be difficult or even impossible to produce using conventional methods. However, the field still faces several practical challenges before these technologies can be used more widely and reliably in industry.

One of the biggest challenges is understanding how processing conditions affect the final properties of a material. In AM, small changes in laser power, scan speed, powder quality, or cooling rate can influence porosity, cracking, residual stress, grain structure, and mechanical performance. This is especially important for titanium alloys, where the relationship between process conditions, defects, microstructure, and properties is highly complex [25]. Similar issues are seen in laser powder-bed fusion of nickel aluminum bronze and GH3230 superalloy. Green-laser processing has shown that ductility can be improved without causing a major loss of strength [26], while laser beam shaping can help control cracking and microstructural development in difficult-to-process superalloys [27]. Future manufacturing systems will need better in situ monitoring and closed-loop control so that process parameters can be adjusted during production rather than only after defects have already formed [7].

Feedstock quality is another major concern. Powder-bed fusion usually depends on highly spherical powders with controlled particle sizes, but these powders are expensive to produce. Using non-spherical powders may reduce cost and improve material efficiency, although irregular particles can create problems with powder flow, packing density, and layer consistency [28].

Ceramic manufacturing also remains difficult because of cracking, shrinkage, debinding, and the high energy required for sintering [29]. Microwave sintering of binder-jetted alumina offers a potentially more energy-efficient approach [30]. Camphor-assisted vat photopolymerization may also help speed up the debinding and sintering of alumina parts [31]. In addition, flash sintering of titanium carbide shows the potential of rapid, field-assisted densification [32]. Even so, these methods must still be tested at larger scales, where temperature control, dimensional accuracy, and uniform heating become much harder to maintain.

Joining dissimilar materials is another important research area, particularly for lightweight and multimaterial structures [33]. Aluminum and steel, for example, are difficult to join because they have very different thermal and metallurgical properties. Magnetic-field-assisted gas metal arc welding has shown that external fields can influence metal flow and improve aluminum-to-galvanized-steel joints [34]. Numerical modeling of aluminum MIG welding can also improve understanding of wire melting, short-circuit transfer, and droplet behavior [35]. For laminated materials, engineered interlocking structures have been used to improve bonding between CuAl and Fe layers [36].

Another clear direction is the integration of manufacturing with post-processing and surface modification. Thermomechanical treatment can significantly change the strength, ductility, and microstructure of additively manufactured 316L stainless steel [37]. Laser-assisted processing of bioactive glasses also shows how manufacturing can be combined with surface modification for biomedical applications [38].

All in all, the future of advanced manufacturing will depend on making these processes more consistent, energy-efficient, scalable, and easier to control. The main challenge is no longer simply proving that a complex part can be made. It is proving that it can be produced repeatedly, affordably, sustainably, and with predictable performance.

## 3. An Overview of the Selected Articles

A major theme of this collection is the expansion of metal AM from shape fabrication toward materials and property control. Park et al. demonstrated direct energy deposition processing of 5 wt% Cr cold-work tool steel, showing that post-heat treatment transformed the as-built microstructure into martensite with fine carbides and markedly improved tensile strength [39]. In a related contribution, Rothen-Chaja et al. examined H13 tool steel produced by LENS^®^ under different in situ thermal exposure conditions during and after deposition, showing that thermal management can be beneficial but may also promote brittleness if phase transformation and carbide precipitation are not properly controlled [40]. Collectively, these studies demonstrate that additive manufacturing of tool steels requires both crack-free deposition and precise control of martensitic transformation, tempering, residual stresses, and toughness.

The selected papers also address difficult-to-process alloys in LPBF, and electron-beam AM. Zhu et al. used quasi-in situ EBSD to reveal variant-dependent slip and twinning in HIP-treated SEBM TA15 titanium alloy, providing a mechanistic basis for texture and performance improvement in additively manufactured titanium components [41]. The work on WE43 magnesium alloy broadened the LPBF processing window by showing that high density can be obtained at elevated laser power and scanning speed, while corrosion in 3.5 wt.% NaCl depends on both porosity and microstructure once high densification is achieved [42]. Uba et al. proposed a CALPHAD-guided strategy for Al 7xxx alloys, combining grain refinement with eutectic solidification to reduce hot cracking and porosity during LPBF [43].

Extending this discussion from processing to durability and certification, Peng et al. compared naturally occurring three-dimensional crack growth in LPBF Scalmalloy^®^ and pre-corroded 7085-T7452, carrying implications for additively manufactured limited-life replacement parts [44]. These experimentally driven studies are complemented by the increasing use of digital tools for process design. Jang et al. developed regression-based machine learning models to predict LPBF melt pool width and depth in Fe-Si alloys, identified input energy as the dominant variable via SHAP analysis, and converted the predictions into power–velocity process maps, which were validated by X-ray computed tomography [45]. This study shows that machine learning can complement experimental and simulation approaches by accelerating process-window identification, particularly for alloys in which melt-pool stability is closely linked to density and functional performance.

Beyond metal-focused AM studies, the collection also demonstrates the rapid development of ceramic and hybrid manufacturing. Freitas et al. reviewed hybrid manufacturing routes that integrate additive and subtractive operations in a single setup, stressing their ability to combine geometric freedom with dimensional accuracy and surface quality [46]. In ceramics, Przybyla et al. showed that robocasting/direct ink writing can produce Al_2_O_3_ oxide ceramic components with promising density, hardness, and bending strength, as well as microstructural homogeneity [47]. Complementarily, Poplawski investigated SLA/DLP-based ceramic printing and demonstrated that optimized geometry, layer thickness, sintering temperature, holding time, and porosity control may considerably improve the mechanical reliability of ceramic parts fabricated by AM [48].

The selected articles similarly broaden the scope of manufacturing systems to include interface- and surface-dominated processes, such as joining, laser-assisted nanoscale bonding, tribology, and precision finishing. Monajati et al. synthesized the mechanical behavior of adhesively bonded joints under tensile loading, compared single-lap, single-strap, and double-strap configurations, and discussed modeling strategies such as cohesive zone modeling, extended finite element methods, and virtual crack closure techniques [49]. Feng et al. showed that femtosecond laser irradiation can generate localized plasmonic heating, mechanical bonding, and large reductions in electrical resistance at Ag nanowire–graphene oxide nanoscale interfaces [50]. Antony Jose et al. connected MXene manufacturing routes, surface functionalization, and tribological behavior, highlighting the potential of two-dimensional carbides and nitrides as solid lubricants or additives while identifying oxidation and long-term stability as key barriers [51]. Yun and Lim linked grinding-wheel bond elasticity, wafer fracture toughness, diamond protrusion, roughness, forces, debris, and subsurface damage, providing practical guidance for high-throughput back grinding of Si, GaP, sapphire, and SiC wafers [52].

## 4. Conclusions

Collectively, these Editor’s Choice Articles suggest that simultaneous control of process physics, material response, geometry, surface condition, and service performance is becoming a defining feature of contemporary manufacturing research. The metal AM contributions highlight the necessity of designing alloys, thermal histories, and post-processing routes in an integrated approach, rather than treating processing and heat treatment as separate steps. The ceramic, hybrid joining, tribology, and precision grinding studies underscore that manufacturing systems must deliver dimensional accuracy, interface reliability, surface integrity, and functional performance.

Another unifying feature is the increasing use of modeling, CALPHAD, machine learning, EBSD, X-ray computed tomography, and fracture mechanics as tools for interpreting and qualifying manufacturing routes. Future advancements are likely to depend on the integration of experimental characterization, predictive simulations, in situ monitoring, and adaptive control, thereby facilitating the transition of advanced manufacturing from process feasibility to reliable industrial implementation.
